# Supplementary material for: Aerosol-induced intensification of cooling effect of clouds during Indian summer monsoon
Source: Nat Commun. 2018 Sep 14;9:3754. doi: 10.1038/s41467-018-06015-5 (PMC6138698; doi:10.1038/s41467-018-06015-5)
Supplement: Supplementary file 1 — Supplementary Information [file 41467_2018_6015_MOESM1_ESM.pdf]

Supplementary Information:

“Aerosol-induced intensification of cooling effect of clouds during Indian summer monsoon”

Supplementary Table 1. Summary of datasets used in this study. All datasets are obtained at a spatial resolution of $1^o \times 1^o$ grid.

Data source and version	Period	Temporal resolution	Data Access Link
MODIS-AQUA L3	2002-2016	Daily, 13:30 local crossing time	https://ladsweb.modaps.eosdis.nasa.gov/
MERRA-2 (V5.12.4)	2002-2016	Daily, 11:30– 14:30 local time	https://disc.sci.gsfc.nasa.gov/daac-bin/FTPSubset2.pl
CERES L3 SYN1deg (Edition 3A)	2002-2016	Daily, 11:30– 14:30 local time	https://ceres.larc.nasa.gov/order_data.php
NOAA-NCEP GDAS (GDAS_0ZF)	2002-2016	Daily, 11:30 local time	https://ladsweb.modaps.eosdis.nasa.gov/
CloudSat (2B-CWC-RVOD.P_R04)	2006-2016	13:30 local time	http://www.cloudsat.cira.colostate.edu/order-data

Supplementary Table 2. Multiple linear regression analysis of the influence of meteorological parameters on the CF, SWCRF, LWCRF, NETCRF, and all-sky albedo analysed over ISMReg. The multiple linear regression analysis carried for 3.5 months, mid-June through October, during each year and the table shows the average and variability among the 3.5 months over 15 years. The parameters influencing the cloud fraction are ordered by the order of significance based on the correlation with the cloud fraction.

Parameter	Regression Slope				
	CF	SWCRF	LWCRF	NETCRF	$A_{All\text{-}Sky}$
MODIS AOD	0.24±0.02	-39.1±4.8	18.8±1.5	-20.95±3.84	0.07±0.01
Temperature at 850 hPa	0.025±0.01	1.0±0.9	0.3±0.3	1.35±0.84	0.003±0.001
RH at 850 hpa	0.009±0.001	-1.8±0.1	0.2±0.03	-1.59±0.08	0.001±0.007
WS between 300 and 500 hPa	0.007±0.008	-1.6±1.1	-0.4±0.4	-1.79±0.81	0.003±0.001
GH at 850 hPa	-0.003±0.001	0.7±0.1	-0.1±0.02	0.61±0.04	-0.001±0.0001
CAPE (10^{-3})	0.024±0.013	-0.7±0.1	3.2±0.7	2.33±1.35	-0.006±0.002

RH: Relative Humidity; WS: Wind Shear; GH: Geopotential Height; CAPE: Convective Available Potential Energy.

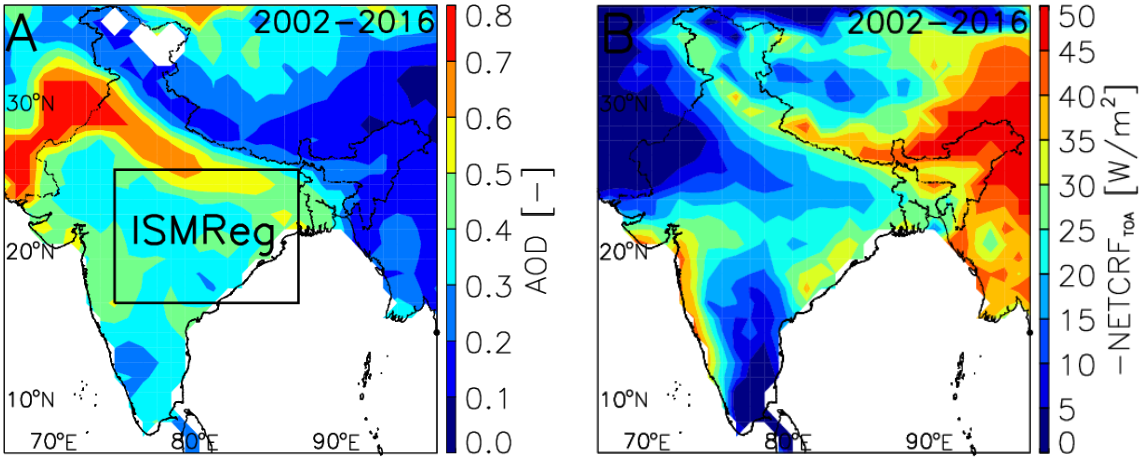

Supplementary Figure 1. Spatial distribution of climatological mean of A) AOD and B) daily mean $NETCRF_{TOA}$ over years 2002-2016. The bounded black box shows the Indian Summer Monsoon region (ISMReg).

Supplementary Note 1.

We have examined variations in CER with CTP (and CER_{ICE} with height) for different aerosol loading conditions using MODIS (and CLOUDSAT) observations to illustrate aerosol-induced micro-physical effect over the IMSReg (Fig.S2). At any pressure level in the troposphere, CER values are found to decrease with increase in aerosol loading (Fig.S2A).Similarly, for any particular CER value (e.g.15 µm) the color changes from blue to cyan to green to yellow and red in the same order indicating that the clouds formed in low AOD scenario (blue color) have higher CER values at lower heights compared to the clouds forming in high AOD scenarios. In convective clouds CER increases with height so composite CTP-CER associations from MODIS observations can be assumed to be similar to CER profiles obtained from a space-time evolving individual clouds[1]. Thus, increase in CER with height is slower for high AOD scenario compared to lower aerosol loading (Fig. S2A). Modeling studies have documented that AOD-associated reduction in the size of cloud droplets may result in the lower gradient of CER due to the associated reduction in collision and coalescence efficiency[2]. The slower increase in cloud droplet size with height leads to reduction in removal of cloud droplets by rain washout and presence of small sized supercooled cloud droplets even above the freezing level. This results in increase in cloud water content both below and above the freezing level and eventually the formation of more ice hydrometeors under high AOD scenario (Fig. S2B). Enhanced formation of ice particles releases a higher amount of latent heat above freezing level and can invigorate the updrafts further resulting in increase in overall cloudiness, cloud depth under high aerosol loading. The ice particles fall from a comparatively high altitude under AIvE causing more rimming and accretion. So the effective radius of ice may be higher with AOD over this region (as seen in Fig. S2C). Along with macro-level changes like the increase in CF and CTP with AOD loading (Fig.2), these microphysical changes are consistent with the AIvE theory over the IMSReg. These microphysical relationships are robustly observed for clouds at all values of CTP. Similar relationships are also evident for MERRA-2 estimated AOD (Fig. S2D-F). Therefore, the brightening of clouds and the positive association between AOD and |CRF| may be linked to AIvE in clouds during ISM. This argument is also substantiated by the close spatial agreement seen between aerosol-associated micro-physical changes and aerosol-associated changes in CRF over the IMSReg (Fig. S1).

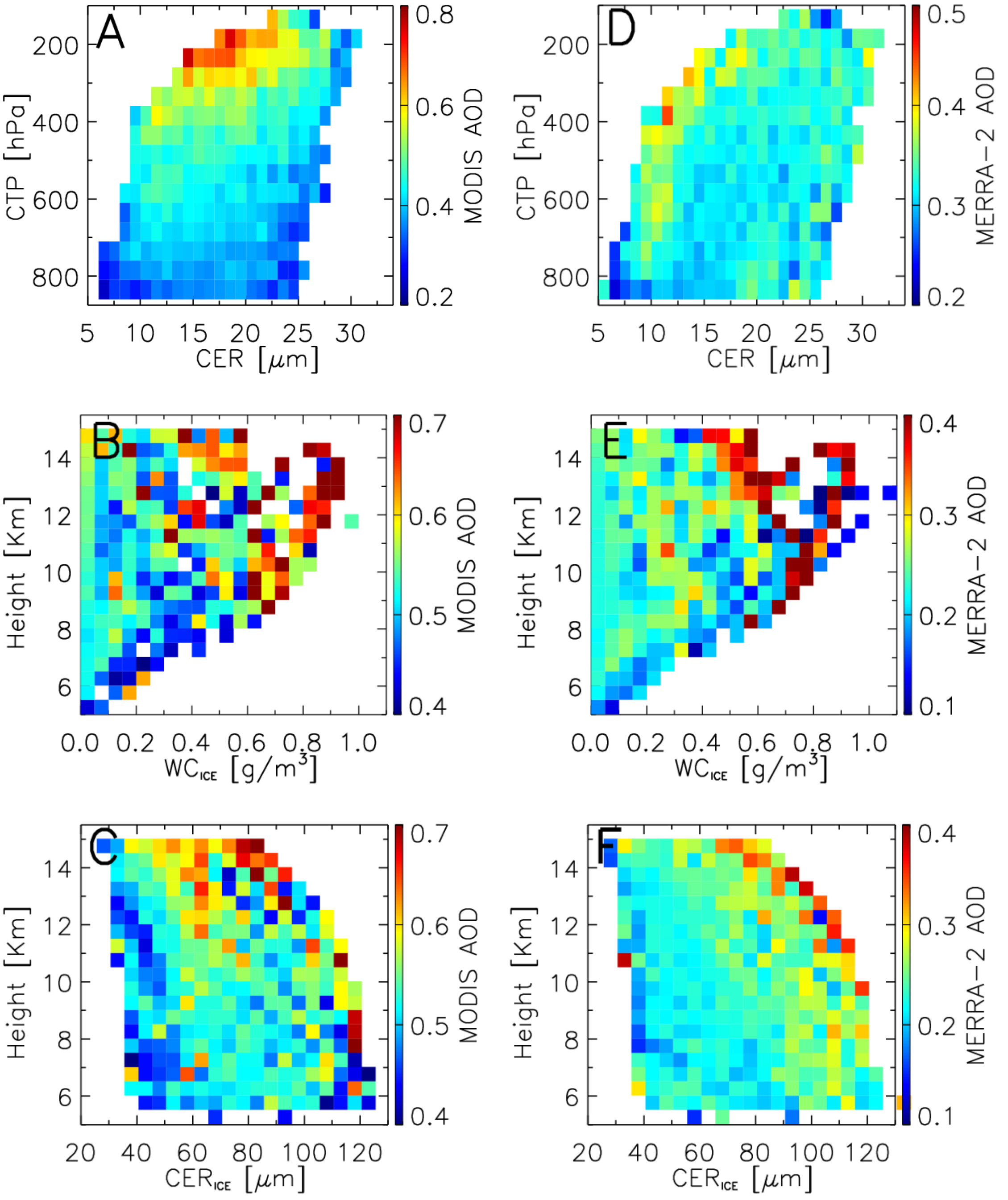

Supplementary Figure 2. Substantiating aerosol-induced cloud invigoration over the Indian Summer Monsoon region. Associations Changes in MODIS CER as a function of CTP and A) MODIS AOD and D) MERRA-2 AOD. Changes in CloudSat WC_{ICE} as a function of CTP and B) MODIS AOD and E) MERRA-2 AOD. Changes in CloudSat CER_{ICE} as a function of CTP and C) MODIS AOD and F) MERRA-2 AOD.

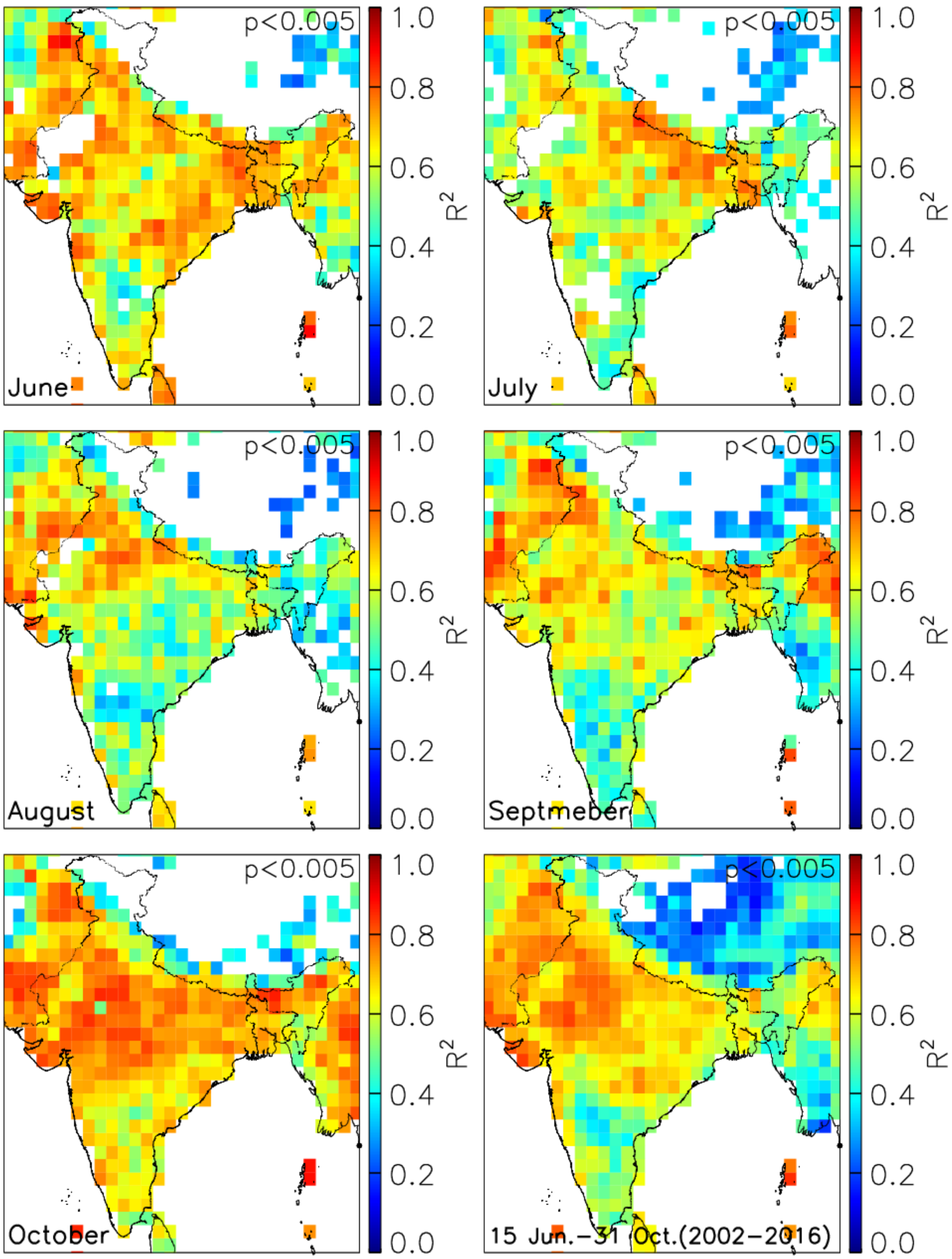

Supplementary Figure 3. Monthly averaged spatial distribution of correlation coefficient (R^2) between MODIS and MEERA-2 AOD for June to October individually as well as averaged over June through October for2002-2016. Statistical student's t-test was performed to test significance of correlation coefficient with a p-value < 0.0005. Note that data points over water bodies (Arabian Sea, Bay of Bengal and Indian Ocean) are not plotted.

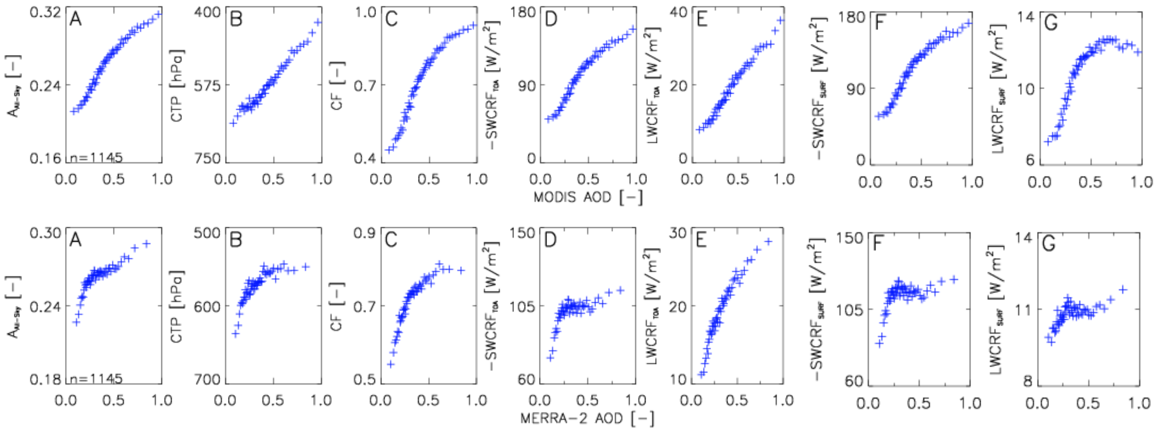

Supplementary Figure 4. Association of MODIS AOD (Top Panel) and MERRA-2 AOD (Bottom Panel) with A) All-Sky Albedo, B) CTP, C) CF, D) $SWCRF_{TOA}$, E) $LWCRF_{TOA}$, F) $SWCRF_{SURF}$ and G) $LWCRF_{SURF}$.

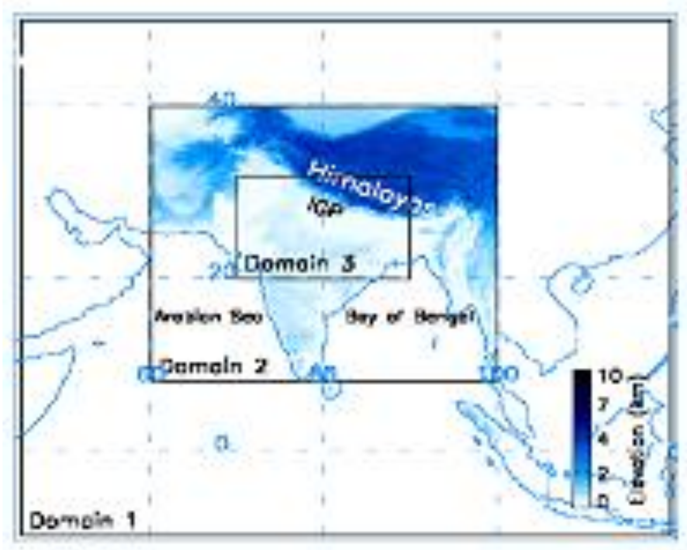

Supplementary Figure 5. Nested domain used for WRF simulation.

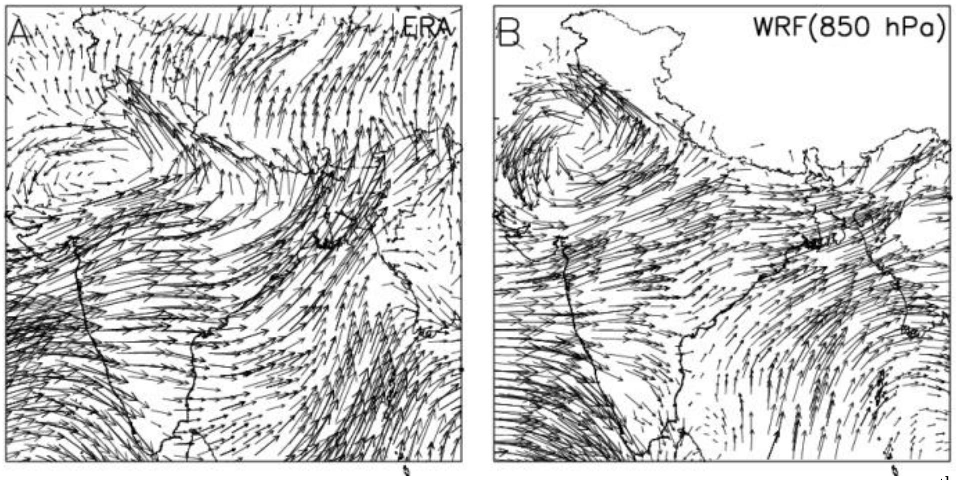

Supplementary Figure 6. ERA interim and WRF wind-vectors during 12th - 17th August, 2011.

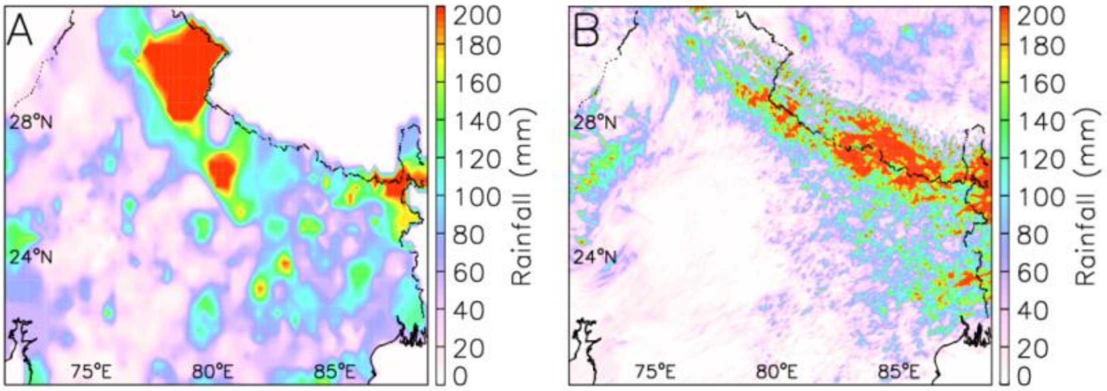

Supplementary Figure 7. Domain- and time-averaged spatial distribution of accumulated rainfall from A) IMD observation and B) WRF simulation over 12th-17th, August 2011.

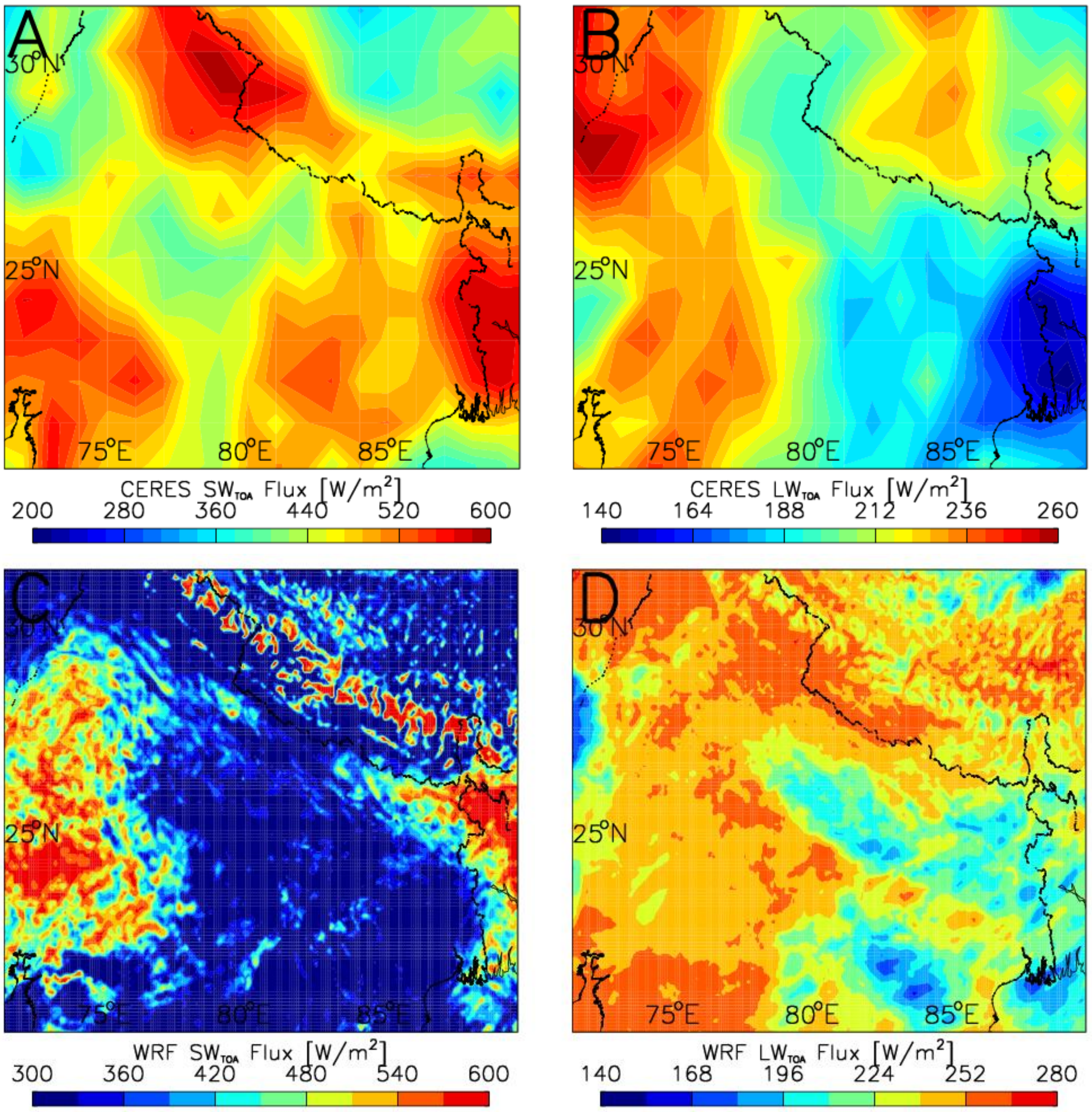

Supplementary Figure 8. Comparison between CERES (A and B) and WRF (C and D) outgoing shortwave and longwave fluxes at the top of the atmosphere, respectively, for 12-17 August 2011.

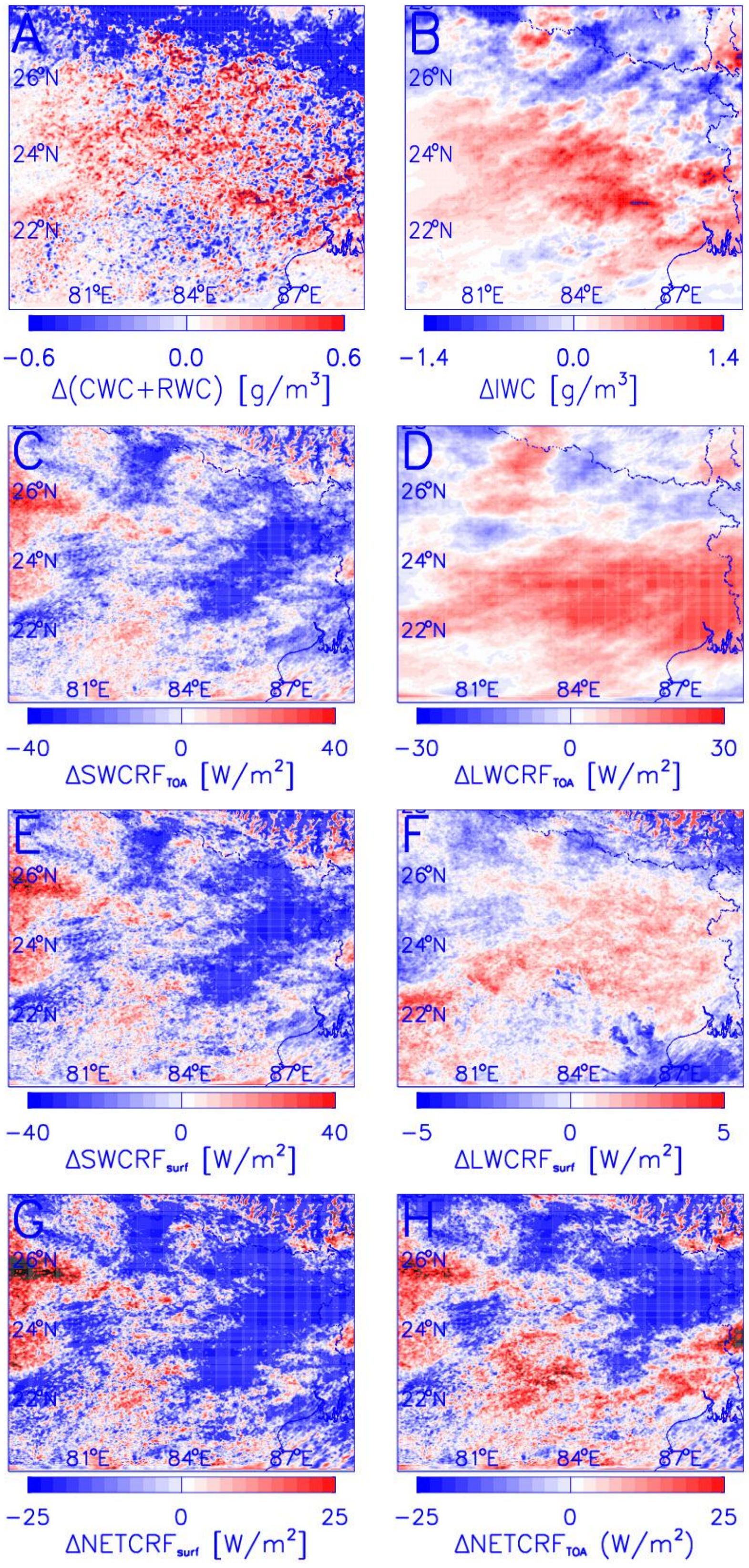

Supplementary Figure 9. WRF Domain- and time-averaged spatial distribution of differences (high CCN –low CCN) in A) CWC+RWC, B) IWC, C) $SWCRF_{TOA}$, D) $LWCRF_{TOA}$, E) $NETCRF_{TOA}$, F) $SWCRF_{SURF}$, G) $LWCRF_{SURF}$ and H) $NETCRF_{SURF}$ during 12th-17th, August 2011.

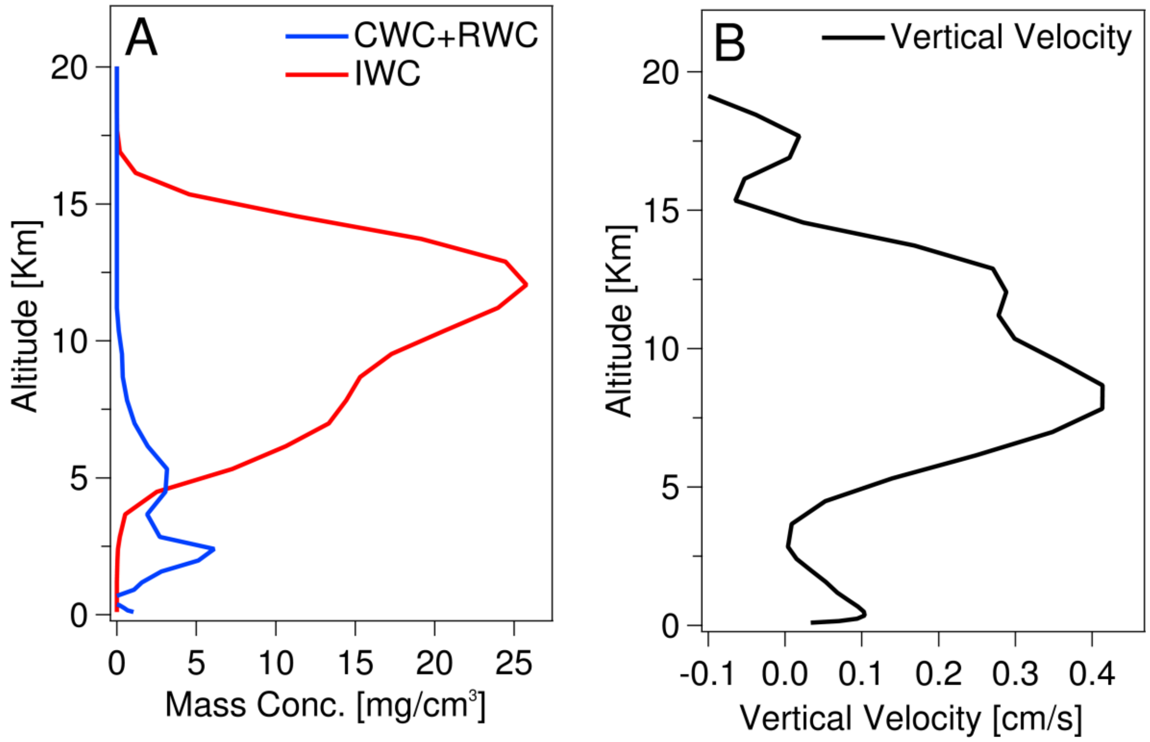

Supplementary Figure 10. Domain- and time-averaged vertical profile differences (high CCN – low CCN) in A) mass concentration of various cloud species and B) vertical wind velocity during12th-17th, August 2011.

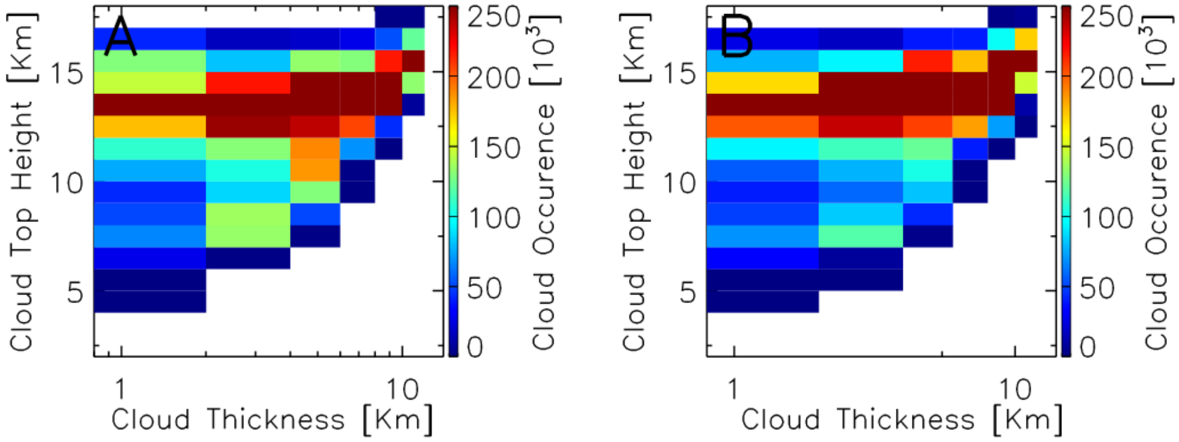

Supplementary Figure 11. Cloud occurrence frequency as a function of cloud thickness and cloud top height for (A) low CCN and (B) high CCN WRF model simulations.

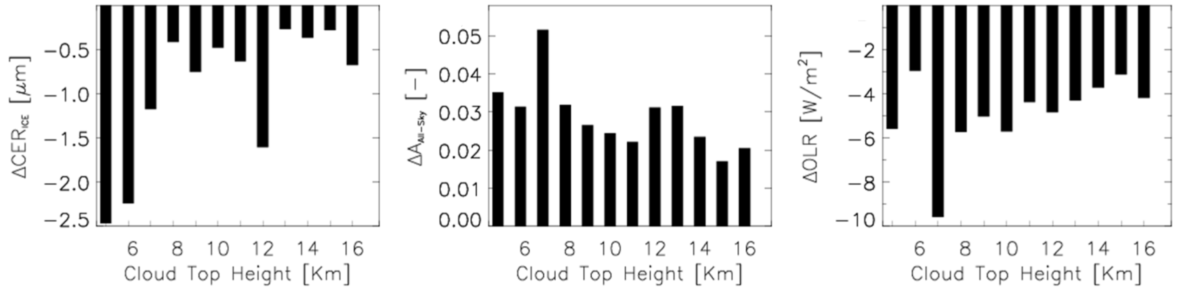

Supplementary Figure 12. Differences (high-low CCN) in A) CER_{ICE}, B) $A_{All\text{-}Sky}$, and C) OLR as a function of cloud top height. The collocated data are binned in 2 km of cloud thickness and 1 km of cloud top height. Then, the CER_{ICE}, $A_{All\text{-}Sky}$ and OLR data values are averaged in each 2 km × 1 km grid cell separately for low CCN and high CCN runs. For each cloud top height bin, all cloud thickness bins are averaged for low and high CCN simulation separately and the difference between them is plotted.

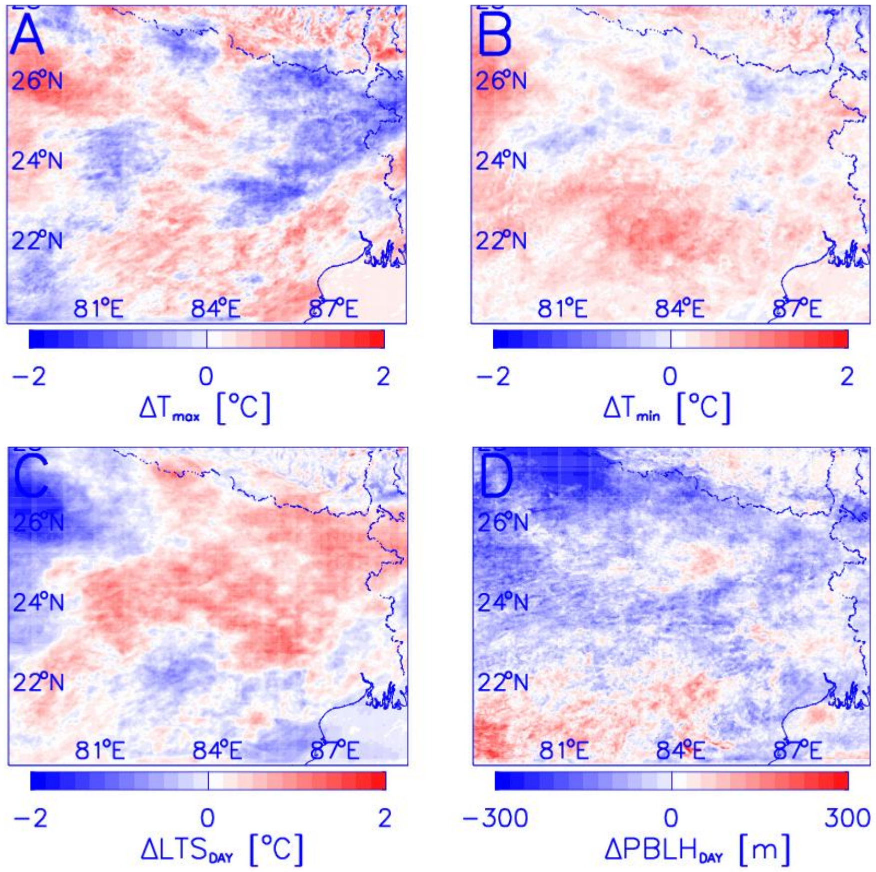

Supplementary Figure 13. Domain- and time-averaged spatial distribution of differences (high CCN – low CCN) in A) T_{max}, B) T_{min}, C) daytime lower tropospheric stability, and D) daytime planetary boundary layer height during 12th- 17th, August 2011.

References

1. Williams E, *et al.* Contrasting convective regimes over the Amazon: Implications for cloud electrification. *J. Geophys. Res. Atmos.* **107**, 8082 (2002).

2. Tao W-K, Chen J-P, Li Z, Wang C, Zhang C. Impact of aerosols on convective clouds and precipitation. *Rev. Geophys.* **50**, RG2001 (2012).
